# Genome-wide Screening of the *Escherichia coli* Keio Knockout Collection Identifies Genetic Determinants of Epetraborole Hypersusceptibility

**DOI:** 10.1007/s10096-025-05183-9

**Published:** 2025-06-13

**Authors:** Anara Babayeva, Bekir Çöl

**Affiliations:** 1 Graduate School of Natural and Applied Sciences, Department of Biology, Muğla Sıtkı Koçman University, Muğla, Turkey; 2 College of Sciences, Department of Biology, Division of Biotechnology, Muğla Sıtkı Koçman University, Muğla, Turkey; 3 Biotechnology Research Center, Muğla Sıtkı Koçman University, Muğla, Turkey

**Keywords:** Epetraborole, *Escherichia coli*, Keio collection, Antibiotic resistance, Gene

## Abstract

**Purpose:**

The escalating threat of antibiotic resistance necessitates innovative strategies to enhance the efficacy of emerging antimicrobials. Epetraborole (EP) is a boron-containing antibiotic targeting leucyl-tRNA synthetase (LeuRS) and has attracted interest for its novel mechanism of action and potential to treat infections caused by multidrug-resistant (MDR) Gram-negative pathogens.

**Methods:**

To elucidate the genetic determinants of EP susceptibility, we conducted a genome-wide screen (GWS) of the *Escherichia coli* Keio knockout collection, which comprises ~4,000 single-gene deletion mutants. Mutants exhibiting increased susceptibility to epetraborole were identified and validated via complementation assays.

**Results:**

Disrupted genes included those involved in leucine biosynthesis (*leuD*), RNA turnover (*rnb*), tRNA modification (*trmU*), ubiquinone biosynthesis (*ubiG*), NAD salvage pathway (*pncA*), arginine transport (*artJ*), transcriptional regulator (*yddM*) and ribosome biogenesis (*yhbY*), suggesting that epetraborole’s primary inhibition of LeuRS synergizes with defects in these pathways. Bioinformatic analyses (Omics Dashboard, DAVID, STRING) linked these genes to tRNA homeostasis, stress response networks, and central dogma processes, implicating tRNA dysregulation as a critical vulnerability under epetraborole-induced stress.

**Conclusion:**

This study identifies novel genetic contributors to epetraborole susceptibility and provides a framework for exploring adjuvant therapies or resistance mechanisms to enhance its clinical utility against MDR infections.

**Supplementary Information:**

The online version contains supplementary material available at 10.1007/s10096-025-05183-9.

## Introduction

The rise of multidrug-resistant (MDR) pathogens has intensified efforts to develop antibiotics with novel mechanisms of action. Boron-containing compounds, long overlooked in antimicrobial drug discovery, have emerged as a promising class due to their unique chemistry and ability to target essential bacterial pathways. Early examples, such as boromycin and tartrolone, demonstrated the potential of boron as a pharmacophore [1–4]. In recent years, this potential has been clinically realized with agents such as tavaborole (a topical antifungal targeting leucyl-tRNA synthetase, LeuRS) [5], epetraborole (EP), a LeuRS inhibitor active against Gram-negative bacteria [5–7] and AN2690 against *Streptococcus pneumoniae* [5–7]. Epetraborole, in particular, represents a breakthrough: its boron-based structure enables precise binding to LeuRS, disrupting tRNA charging and protein synthesis [8].

While conventional antibiotics target pathways such as cell wall synthesis (e.g., β-lactams) or ribosome function (e.g., macrolides), epetraborole’s inhibition of LeuRS offers a distinct mechanism to circumvent existing resistance [9, 10]. Preclinical studies highlight its broad-spectrum activity, showing efficacy against *Mycobacterium abscessus* (including drug-resistant variants), Chlamydia, and cystic fibrosis-associated pathogens [11–14]. Notably, synergy with metabolites like norvaline suggests potential for combination therapies [15]. However, despite these advances, the genetic determinants of bacterial susceptibility to epetraborole remain poorly characterized. Identification of these factors is critical for optimizing its use, predicting resistance, and developing adjuvants.

Here, we address this gap by performing a genome-wide screen (GWS) of the *Escherichia coli* (*E. coli*) Keio knockout collection. By systematically interrogating ~4,000 mutants, we identify genes and pathways that modulate epetraborole susceptibility, providing mechanistic insights into its activity and vulnerabilities in bacterial stress response networks.

## Materials and methods

### Bacterial strains and growth media

The *Escherichia coli* Keio knockout collection (parental strain BW25113) was used for genome-wide screening (GWS). This collection comprises 3,985 non-essential gene deletion mutants, each replaced with a kanamycin resistance cassette flanked by FLP recombination target (FRT) sites [16]. Glycerol stocks of the mutants were stored in 47 96-well microplates at −80°C. Cultures were grown in lysogeny broth (LB) medium (10 g/L tryptone, 5 g/L yeast extract, 10 g/L NaCl) supplemented with 15 g/L agar for solid media. Kanamycin (50 μg/mL) or chloramphenicol (30 μg/mL) was added as needed.

### Determination of Epetraborole Antibacterial Activity

To establish optimal screening concentrations, spot tests were performed on wild-type *E. coli* BW25113 and 20 randomly selected Keio mutants. Strains were streaked from −80°C stocks onto LB agar and incubated overnight at 37°C. Single colonies were inoculated into 5 mL LB and grown to stationary phase (16 h, 37 °C, 200 rpm). Cells were pelleted, washed with phosphate-buffered saline (PBS), and adjusted to OD_600_ of 0.5. Serial 2-fold dilutions (1:1 to 1:16) were spotted onto LB agar containing epetraborole (EP; MedKoo Biosciences, Cat#319569) at concentrations of 0, 0.5, 1, 2, 3, 4, 5, and 6 μg/mL (dissolved in DMSO). Plates were incubated at 37 °C for 5 days, with daily imaging using a digital camera. Susceptibility was quantified by comparing growth inhibition across dilutions.

### Genome-wide Screening

Mutants were inoculated from glycerol stocks into 96-well plates containing 200 μL LB per well using a microplate replicator (Boekel Scientific, USA). After overnight growth (37°C), cultures were replicated onto LB-kanamycin (50 μg/mL) plates and incubated until mid-log phase (OD_600_ = 0.2–0.3). Cells were then pinned onto LB agar containing EP (0, 0.5, 1, 2, 3, and 4 μg/mL) in duplicate. Plates were imaged daily over 5 days. Mutants were classified as hypersusceptible (HS), moderately susceptible (MS), or low susceptible (LS) based on growth inhibition duration and antibiotic concentration (Table 1). Microcolonies or reduced growth observed after Day 1 were documented but did not constitute growth for susceptibility classification. Final phenotypes were determined through replicated spot assays assessing complete inhibition.

**Table 1:** Genome-wide screening of the Keio mutant line reveals 44 mutants susceptible to EP

Keio mutant strains susceptible to EP ^a^	Growth level at day ^b^	Observed susceptibility (μg/ml EP) ^c^
*ΔubiG, ΔpncA, ΔtrmU, ΔartJ, ΔyddM, Δrnb, ΔyhbY, ΔyggG, ΔymjB, ΔybcM, Δwzc, Δfrc, ΔynfB, ΔyafM, ΔcmtA, ΔyjcE, ΔybeU, ΔprpR, ΔyfbV, ΔyhcG, ΔyhbU, ΔydfD, ΔenvZ, ΔyheM, ΔrpmE*	No growth was observed at days 1 to 5	No growth was observed at concentrations of 1,2,3 and 4 μg/ml EP
*ΔyecT, ΔotsA, ΔhslO, ΔtrmC*	No growth at days 1 to 4	
*ΔgapC, ΔcreC, ΔyagP, ΔybgA, ΔpurR, ΔyfaY, Δsmg, ΔcytR*	No growth at days 1 to 3	
*ΔyghU, ΔygjG, ΔyegX, ΔfbaB*	No growth at days 1 to 2	
*ΔoppF, ΔflgG, ΔleuD*	No growth at days 1	
The BW25113 control strain and other mutant strains	are able to grow on all days, with better growth on the later days.	Can grow up to 4 μg/ml EP

^a^ EP-susceptible knockout mutants are indicated by the gene name. The Keio mutant line of *E. coli* contains nearly 4000 non-essential gene knockout mutants, and this line has been screened for susceptibility to epetraborole at sublethal concentrations. The 44 mutants listed here were found to be susceptible to EP at a certain sublethal concentration. These mutants were later tested for tolerance to EP using the sequential spot assays listed in the next table.

^b^ Observations were made on days 1, 2, 3, 4, and 5. The growth of the mutants was recorded. Primary susceptibility calls were based strictly on complete growth inhibition at 24hr (no visible colonies). Reduced growth observations served as secondary indicators for spot-test validation.

^c^ LB media with EP concentrations ranging from 0 to 4 μg/ml were used to test the growth of the strains. The growth of the mutants was observed over a period of five days for each concentration of EP.

### Sequential spot tests

To validate antibiotic susceptibility phenotypes, spot assays were performed as described [17]. Briefly, mutant strains were grown in LB broth to mid-exponential phase, normalized to OD_600_ of 0.5, and subjected to serial 2-fold dilutions (1:1 to 1:16). These dilutions were spotted onto LB agar plates containing EP (0, 0.5, 1, 2, 3, and 4 μg/mL). Growth was monitored daily for 5 days, with susceptibility determined by comparing inhibition patterns against the wild-type control to identify hypersusceptible mutants after overnight incubation.

### Complementation of Hypersusceptible Mutants

The plasmids containing the genes of interest were obtained from the ASKA plasmid library [18], which contains 4,327 *E. coli* W3110 open reading frames (ORFs) cloned into pCA24N (chloramphenicol resistance). The plasmids were transformed into the particular HS mutants via CaCl_2_ heat-shock [19, 20]. Transformants were selected on LB-chloramphenicol (30 μg/mL) and validated by repeating EP susceptibility assays under screening conditions.

### Bioinformatics Analysis

Gene functions and corresponding UniProt IDs were annotated using EcoCyc [21], a curated database for *E. coli* K-12 genomics and metabolic pathways. Functional enrichment analysis was performed using DAVID (v6.8) [22] with a false discovery rate (FDR) cutoff of 0.1 to control for multiple testing, and results were filtered at a significance threshold of 2 standard deviations from the mean to identify robust associations. For pathway analysis, we employed the Omics Dashboard within Pathway Tools [21] to map the data onto known metabolic and regulatory pathways. Protein-protein interaction networks were constructed using STRING (v12.0) [23], with interactions limited to those having high-confidence scores (>0.7) to ensure biological relevance. Network analysis revealed key functional modules and potential protein complexes of interest. Evolutionary conservation of susceptibility determinants was assessed through NCBI BLASTP analysis against the non-redundant protein database.

## Results

### Genome-wide Screening Identifies Epetraborole-Hypersusceptible Mutants

**Fig. 1:**
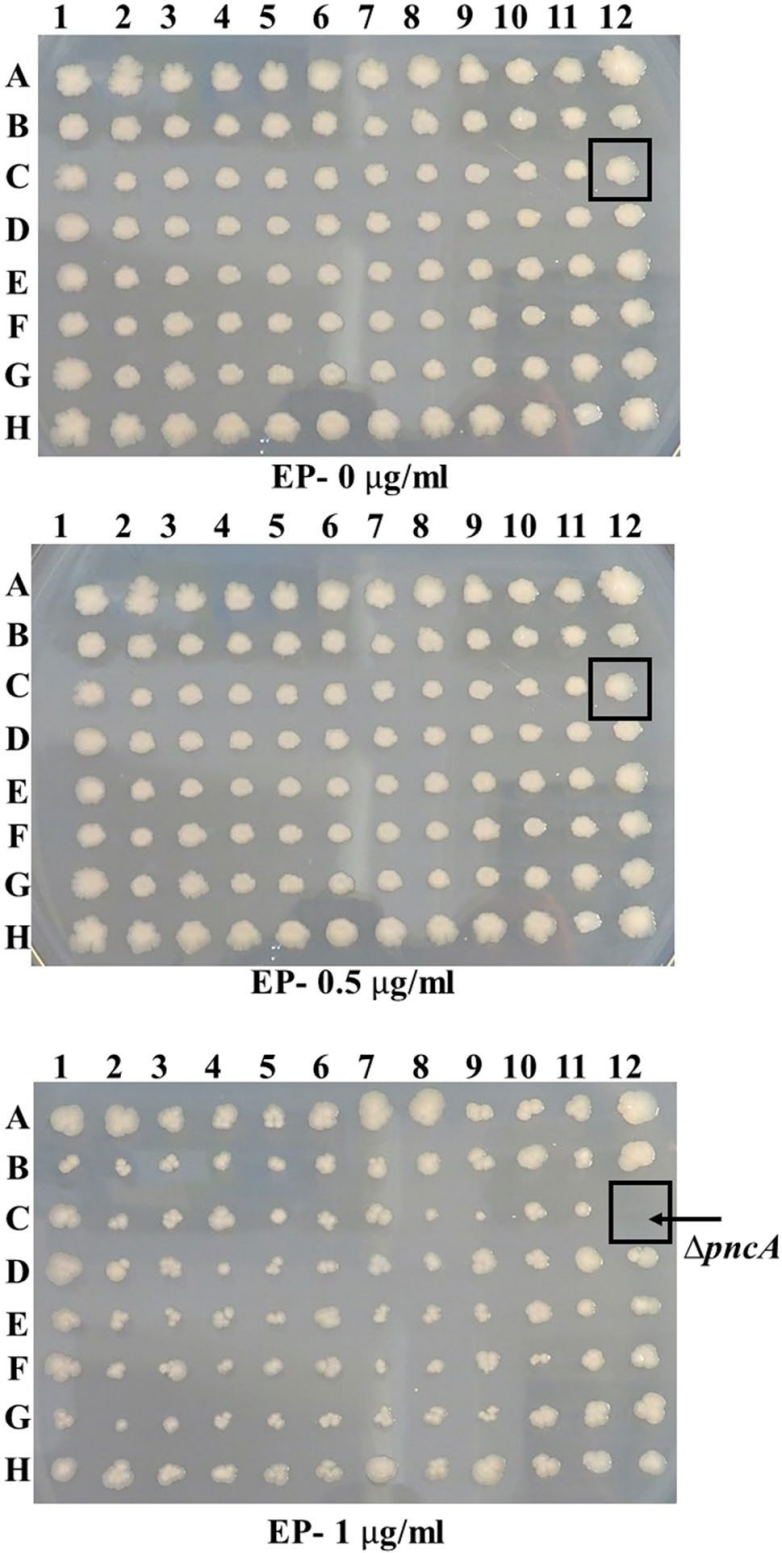
Genome-wide screening identifies EP-susceptible mutants in *E. coli*. A representative genome-wide screen for EP susceptibility is illustrated using the *E. coli* K-12 BW25113 *ΔpncA* mutant. Keio collection microplate 23 (containing *ΔpncA*) was incubated on LB agar supplemented with sublethal concentrations of EP (0–4 μg/ml). Growth inhibition of the *ΔpncA* mutant was observed at ≥1 μg/ml EP (arrow), compared to unaffected surrounding colonies. This screen prioritized mutants for downstream validation (see Methods). Screening plates for all of the EP hypersensitive mutants are given in Figure S1 (Supplementary Material Figure S1)

Genome-wide screens of the *Escherichia coli* Keio collection (3,985 mutants) were conducted on LB agar containing epetraborole (EP; 0, 0.5, 1, 2, 3 and 4 μg/ml). Growth of the mutants was monitored over 5 days, and strains susceptible to EP were identified and listed. An example of the genome-wide screening result is shown in Fig. 1. Additionally, Figure S1 illustrates the GWS results for the hypersusceptible mutants. Forty-four mutants were susceptible to EP at one or more concentrations as a result of GWS experiments. The list of these EP susceptible knockout *E. coli* mutants is presented in Table 1, accompanied by their growth phenotypes and specific observations following the GWS experiments. Mutants were pinned onto EP-containing LB-agar in biological duplicate across two independent screens. Follow-up spot tests and complementation assays were performed in triplicate.

### Evaluation of GWS-identified susceptible mutants using a sequential spot test

**Fig. 2:**
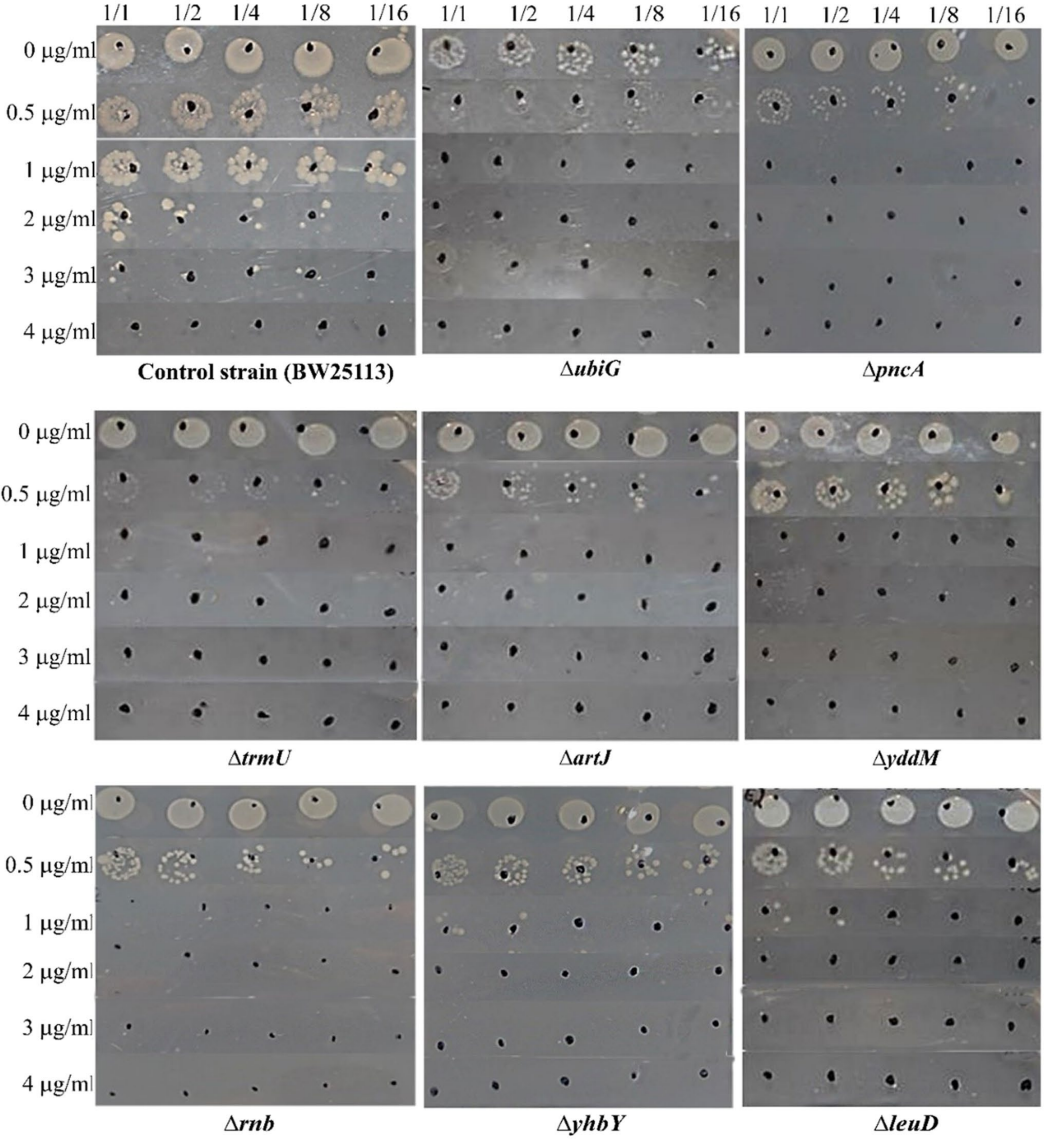
Spot-test confirmation of EP susceptibility in highly susceptible mutants identified through GWS. Spot tests were performed to validate the EP susceptibility of all 44 mutants including eight highly susceptible mutant strains identified via GWS. Serial dilutions (1/1, 1/2, 1/4, 1/8, 1/16) of each strain and the wild-type control (normalized to an OD_600_ of 0.5) were tested against six EP concentrations (0–4 μg/ml). Growth patterns for mutants and the wild-type strain are shown, with strain identifiers labeled below each panel

To confirm the susceptibility of the 44 mutant strains identified as EP-susceptible in the genome-wide screen, sequential spot tests were performed on all mutant strains and the control strain, as outlined in the Materials and Methods section. Strains were tested at EP concentrations of 0, 0.5, 1, 2, 3, and 4 μg/ml. As shown in Fig. 2, mutant strains exhibited complete growth inhibition at EP concentrations ≥1 μg/ml, while the control strain retained viability. Representative spot test results for selected susceptible mutants in EP-containing media are illustrated in Fig. 2. The susceptibility profiles of all 44 mutants, summarized in Table 2, revealed three categories based on growth inhibition thresholds: (1) Highly susceptible (HS; *n*=8): No growth or significantly reduced growth at 1–4 μg/ml EP. (2) Moderately susceptible (MS; *n*=34): No growth or reduced growth at 2–4 μg/ml EP. (3) Low susceptibility (LS; *n*=2): Growth inhibition observed only at 3–4 μg/ml EP.

**Table 2:**
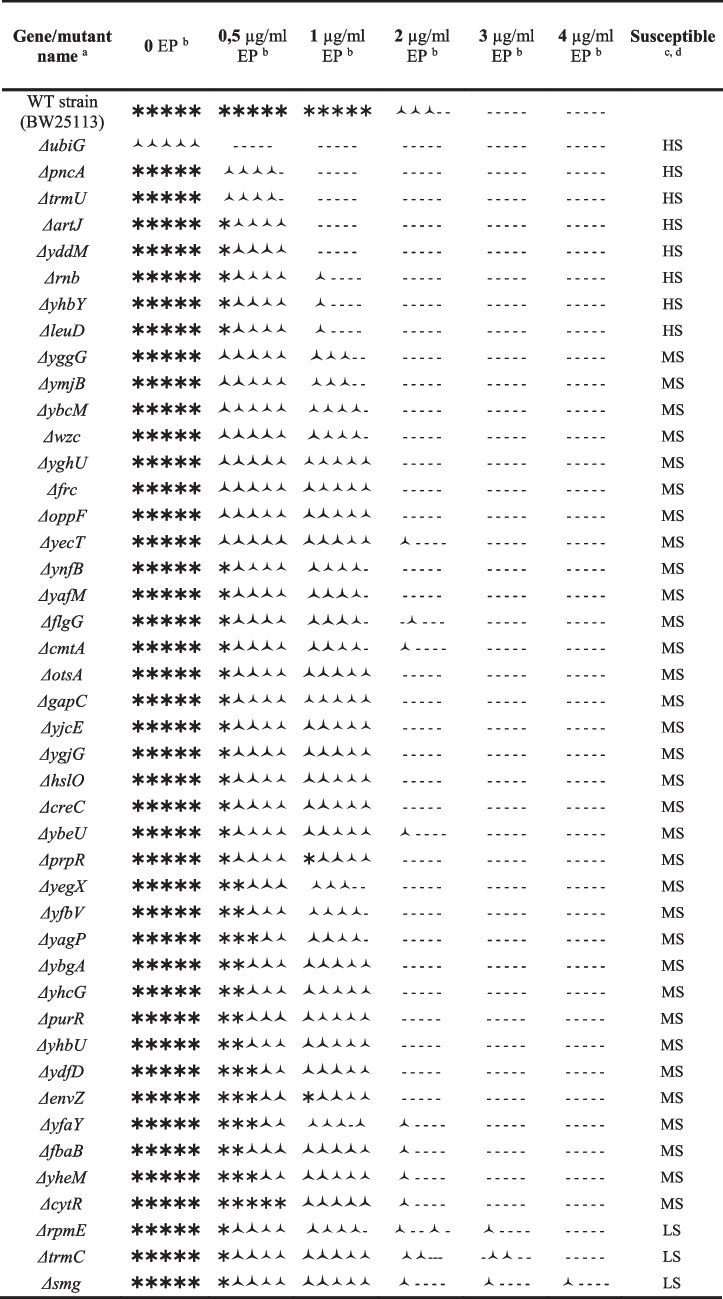
Spot test results to determine EP susceptibility levels of the mutants

^a^ EP-susceptible knockout mutants are designated by their gene symbols (e.g., *ΔgeneX*).

^b^ Strains were tested in LB medium supplemented with EP at concentrations of 0, 0.5, 1, 2, 3, and 4 μg/ml..

^c^ The stars indicate the relative growth of the spotted bacterium strains. Sequential spot tests were performed following the protocol in the Materials and Methods section. Five spots represent the bacterial dilutions, from left to right: OD_600_ 0.5, 0.25, 0.125, 0.063, and 0.031. Full-sized bold stars represent relatively robust growth, smaller stars with fewer edges show relatively reduced growth and dash (-) indicates no growth. Growth after 48 hours were scored.

^d^ The mutants were categorized as highly susceptible (HS; *n*=8), moderately susceptible (MS; *n*=34), or low susceptible (LS; *n*=2) based on their growth levels. HS mutants exhibited no growth or reduced growth at 1–4 μg/ml EP, MS mutants exhibited no growth or reduced growth at 2–4 μg/ml EP and LS mutants showed susceptibility at 3–4 μg/ml EP. Estimated MIC ranges: HS mutants: 0.5–1 μg/ml; MS: 2 μg/ml; LS: 3 μg/ml.

### Complementation of highly susceptible mutants

**Fig. 3:**
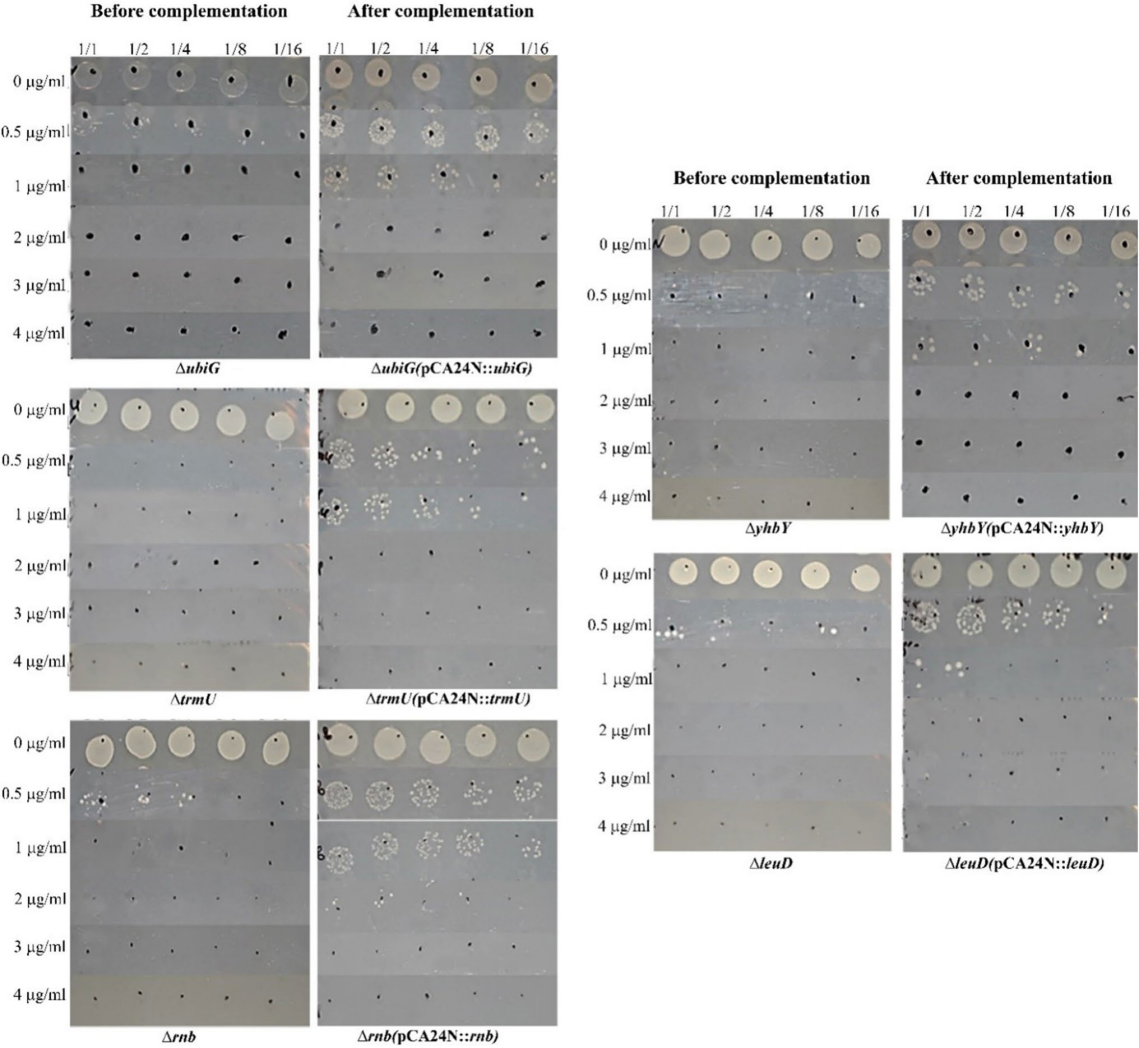
Complementation analysis of EP-susceptible mutants. Growth phenotypes of five gene-knockout mutants (*ΔubiG*, *ΔyhbY*, *Δrnb*, *ΔleuD*, *ΔtrmU*) and their complemented strains were assessed via spot tests in media containing increasing EP concentrations (0–4 μg/ml). Strains were serially diluted (1/1 to 1/16) and spotted onto agar plates as described in Materials and Methods. Complementation with plasmid-borne copies of the deleted genes (pCA24N::gene) restored EP resistance, as evidenced by growth recovery in the presence of sublethal EP concentrations. *ΔubiG* and *ΔyhbY* mutants: Growth restored up to 2 μg/ml EP after complementation (vs. inhibition at 0.5 μg/ml in mutants). *Δrnb* and *ΔleuD* mutants: Resistance increased to 3 μg/ml and 4 μg/ml EP, respectively, upon gene reintroduction (vs. inhibition at 1 μg/ml in mutants). *ΔtrmU* mutant: Complementation rescued growth up to 2 μg/ml EP (vs. no growth at 0.5 μg/ml in the mutant)

To confirm that EP susceptibility in the most susceptible mutants resulted from the targeted gene deletions, complementation experiments were performed using the *ΔubiG*, *ΔpncA*, *ΔtrmU*, *ΔartJ*, *Δrnb*, *ΔyhbY*, and *ΔleuD* strains. Plasmids containing the genes of interest (*ubiG*, *pncA*, *trmU*, *artJ*, *rnb*, *yhbY*, *leuD*) from the ASKA plasmid library [18] were transformed into their respective mutant strains. Spot assays revealed that plasmid-mediated expression of *rnb*, *leuD*, *trmU*, *ubiG*, and *yhbY* restored wild-type EP resistance in their corresponding mutants (Fig. 3). Complementation successfully restored EP resistance in *ΔubiG*, *ΔyhbY*, *Δrnb*, *ΔleuD*, and *ΔtrmU* mutants. However, complementation of *ΔpncA* and *ΔartJ* mutants with pCA24N::*pncA* and pCA24N::*artJ*, respectively, failed to restore resistance (Figure S2). Additionally, *ΔyddM* was excluded due to unavailability of its ASKA clone. While parental mutant strains showed growth inhibition at sublethal EP concentrations, complemented strains exhibited restored growth under identical conditions (Fig. 3, Table 3).

**Table 3:** Spot test results of EP-susceptible strains before and after complementation

Strains	Spot test result EP (μg/ml) susceptibility	Condition
*E. coli* K-12 BW25113 ∆***rnb*** (pCA24N)	1 μg/ml	Before complementation
*E. coli* K-12 BW25113 ∆***rnb*** (pCA24N::***rnb***)	3 μg/ml	After complementation
*E. coli* K-12 BW25113 ∆***yhbY*** (pCA24N)	0,5 μg/ml	Before complementation
*E. coli* K-12 BW25113 ∆***yhbY*** (pCA24N::***yhbY***)	1 μg/ml	After complementation
*E. coli* K-12 BW25113 ∆***leuD*** (pCA24N)	1 μg/ml	Before complementation
*E. coli* K-12 BW25113 ∆***leuD*** (pCA24N::***leuD***)	4 μg/ml	After complementation
*E. coli* K-12 BW25113 ∆***trmU*** (pCA24N)	0,5 μg/ml	Before complementation
*E. coli* K-12 BW25113 ∆***trmU*** (pCA24N::***trmU***)	2 μg/ml	After complementation
*E. coli* K-12 BW25113 ∆***ubiG*** (pCA24N)	0,5 μg/ml	Before complementation
*E. coli* K-12 BW25113 ∆***ubiG*** (pCA24N::***ubiG***)	1 μg/ml	After complementation

For the *ΔubiG* and *ΔyhbY* mutants, complete growth inhibition at 0.5 μg/ml EP was seen but complemented strains grew at concentrations up to 2 μg/ml EP. The growth of *Δrnb* and *ΔleuD* mutants ceased at 1 μg/ml EP and complemented strains tolerated up to 3 μg/ml (*rnb*) and 4 μg/ml (*leuD*) EP. *ΔtrmU* mutant showed no growth at 0.5 μg/ml EP; complementation with *trmU* gene restored growth up to 2 μg/ml EP. Therefore, these results confirm that EP susceptibility in these mutants is directly attributable to the deleted genes, ruling out secondary mutations.

### Pathway analysis of EP-susceptible mutant strains

Pathway analysis was performed to identify functional networks and biological processes associated with EP susceptibility, providing mechanistic insights into gene-phenotype relationships. A total of 44 EP-susceptible gene hits were analyzed using pathway-centric tools, including the Omics Dashboard [21], DAVID Bioinformatics’ Fold Enrichment module [22], and STRING DB [23].

**Fig. 4:**
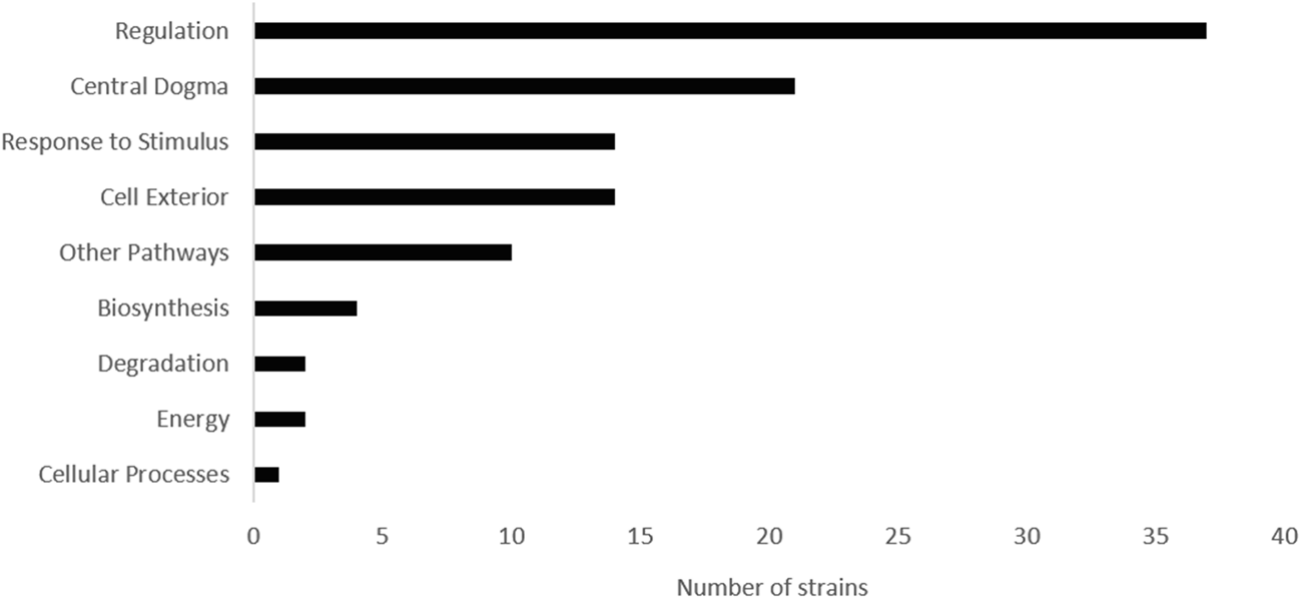
Functional categorization of EP-susceptible genes using Omics Dashboard. Genes linked to EP susceptibility were classified into functional systems via Omics Dashboard and annotated using the EcoCyc database. Numerical values reflect gene counts per system, with several genes mapping to multiple categories. Dominant systems included: Regulation (transcriptional/translational control), Central Dogma (DNA/RNA/protein metabolism), Response to Stimulus (stress adaptation), Cell Exterior (membrane/secretory functions). Underrepresented systems: Biosynthesis, Energy Metabolism, and Degradation pathways (see Supplementary Table for full data)

Omics Dashboard analysis classified these genes according to Systems, Subsystems, and Gene Product Activity and level of susceptibility to EP (Supplementary Table). This analysis revealed that the majority of the genes were classified under the “Regulation”, “Central Dogma”, “Extracellular”, “Response to Stimulus” and “Other Pathways” systems (Fig. 4). Systems such as “Biosynthesis”, “Energy”, “Degradation” and “Cellular Processes” were comparatively less represented (Fig. 4).

**Fig. 5:**
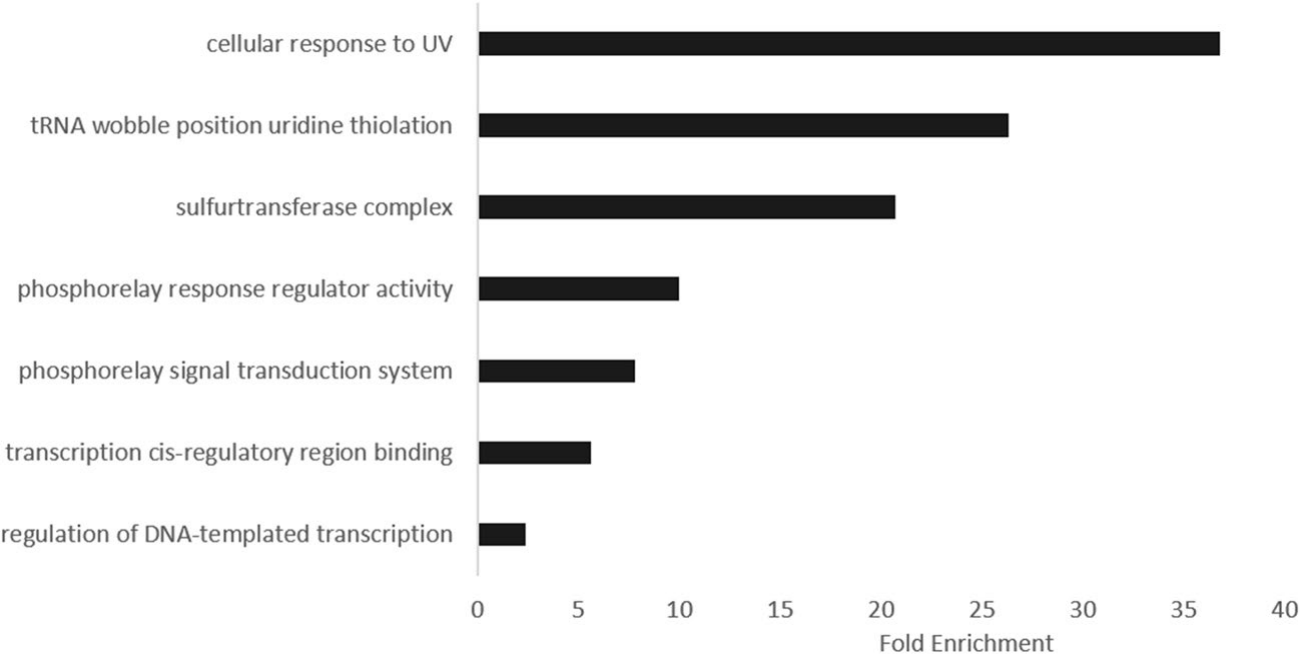
Functional enrichment analysis of EP-susceptible gene hits in *E. coli*. Gene Ontology (GO) enrichment for EP-susceptible gene hits was performed using DAVID (version 6.8; [24]) with the *E. coli* K-12 genome as the background. Clusters containing ≥3 genes and meeting statistical significance (*p* < 0.05) are shown. Enriched functional categories include tRNA wobble uridine thiolation, sulfur transferase complex activity, phosphorelay signaling, and DNA-dependent transcription regulation, highlighting pathways implicated in EP susceptibility

The DAVID Fold Enrichment module [22, 24, 25] was employed to identify enriched biological processes and molecular functions associated with the 44 EP-susceptible gene hits. Gene Ontology (GO) analysis revealed significant enrichment (*p* < 0.05) for processes linked to EP susceptibility, including cellular response to UV (DNA damage repair), tRNA wobble uridine thiolation (tRNA modification), and sulfur transferase complex activity (redox/metabolite regulation). Additionally, EP susceptibility correlated with DNA-dependent transcription regulation (transcriptional control), *cis*-regulatory region binding (promoter/DNA interaction), and phosphorelay signaling and response regulator activity (two-component signaling systems) (Fig. 5). These findings suggest that EP disrupts pathways critical for bacterial stress adaptation, including transcriptional regulation, tRNA fidelity, and redox homeostasis.

**Fig. 6:**
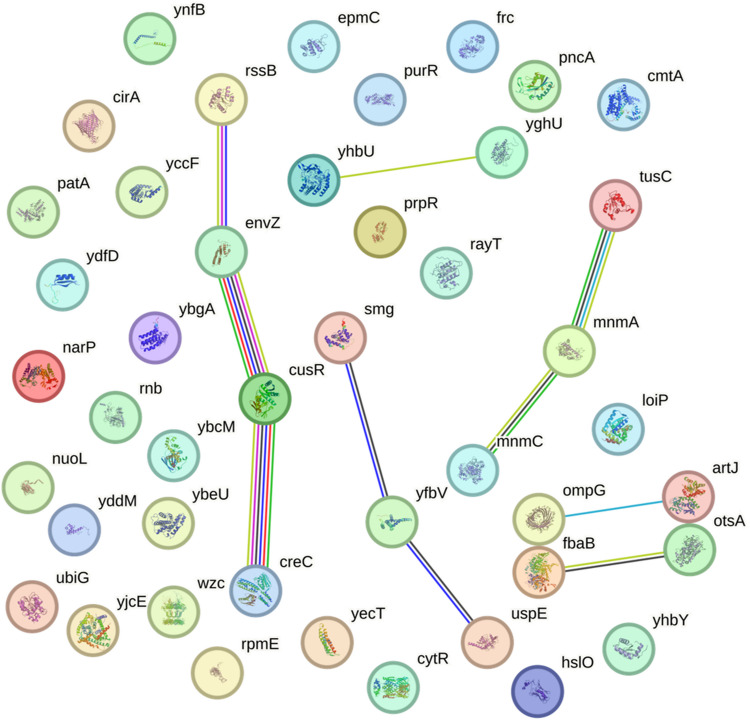
Protein interaction network of EP-susceptible gene hits in *E. coli*. STRING analysis (version 12.0; confidence score ≥0.4) reveals functional associations among genes linked to EP susceptibility in *E. coli* mutants. Nodes represent genes, and edges indicate predicted functional partnerships (e.g., co-expression, shared pathways, physical interactions). The color scheme represents different types of supporting evidence: database annotations (blue), experimentally validated interactions (purple), gene proximity (green), gene fusion events (red), gene co-occurrence/co-evolution (dark blue), text-mining (yellow), co-expression (black) and protein homology (light blue). The network suggests EP disrupts bacterial homeostasis via coordinated pathways. The core interacting susceptibility genes identified by genome-wide screens are: *envZ*, *creC*, *yfbV*, *smg*, *yhbU*, *yghU*, *mnmA* (*trmU*), *artJ*, *fbaB*, and *otsA*. Additional interacting genes (*rssB*, *cusR*, *uspE*, *tusC*, *mnmC*, *ompG*) provide broader biological context for these pathways.

To delineate functional relationships among the 44 EP-susceptible gene hits, protein-protein interaction networks were analyzed using STRING DB analysis [23]. We first analyzed interactions between 44 EP-hypersensitive mutants alone, which showed limited direct connectivity. Therefore, we used the “add more proteins” function of STRING to reveal functional pathways connecting our hits to known interactors. The secondary interaction network (Fig. 6) revealed functionally linked clusters, including stress/toxin response (*rssB* (proteolysis regulation), *envZ* (osmolarity sensing), *cusR* (copper resistance), and *creC* (two-component signaling)), transcriptional regulation (*smg* (small RNA binding), *yfbV* (putative transcriptional regulator), and *uspE* (universal stress protein)), tRNA/modification pathways (*mnmC* (tRNA methylation), *mnmA* (tRNA thiolation), and *tusC* (tRNA transport)) and metabolic/transport systems (*yhbU*/*yghU* (putative ABC transporters), *ompG*/*artJ* (outer membrane pore/arginine transport), and *fbaB*/*otsA* (glycolysis/osmoprotectant synthesis)). These clusters suggest coordinated roles in stress adaptation, transcriptional control, and metabolite transport, aligning with EP’s proposed mechanism of disrupting bacterial homeostasis (Fig. 6). It should be noted that the 10 interacting genes (*envZ*, *creC*, *yfbV*, *smg*, *yhbU*, *yghU*, *mnmA* (*trmU*), *artJ*, *fbaB* and *otsA*) in Fig. 6 were found to be susceptible to EP by genome-wide scanning. The interacting genes other than these genes (*rssB*, *cusR*, *uspE*, *tusC*, *mnmC* and *ompG*) represent genes that allow for broader biological conclusions. Consistent with network analyses in antibiotic studies, this approach helps to explain how separate mutations can converge in common vulnerability pathways.

## Discussion

The growing recognition of boron-containing compounds as biologically and pharmacologically significant agents has positioned boron-based antibiotics, such as epetraborole, as promising candidates for novel antimicrobial therapies. In this study, we employed a genome-wide screening approach to systematically identify genetic determinants of epetraborole (EP) hypersusceptibility. Our findings reveal critical pathways and genes whose disruption amplifies EP susceptibility, shedding light on its mechanism of action and potential bacterial vulnerabilities. Below, we contextualize these results within broader biological frameworks, propose mechanistic hypotheses, and highlight implications for antibiotic development.

The study utilized the Keio collection, a comprehensive library of *E. coli* single-gene knockouts, to screen ~4,000 mutants for EP hypersusceptibility. Initial genome-wide phenotypic screening identified mutants with growth defects under sub-inhibitory EP concentrations. These hits were rigorously validated through dose-response spot assays and complemented via plasmid-mediated gene reintroduction to confirm genotype-phenotype causality. Subsequent bioinformatics analyses (Omics Dashboard, DAVID, STRING) mapped susceptible mutants to cellular systems and pathways, enabling mechanistic hypotheses. This multi-tiered approach ensured robust identification of genes critical to EP susceptibility. It should be emphasized that our genome-wide screen focused exclusively on non-essential genes. Essential genes (e.g., LeuRS itself) likely modulate EP susceptibility but require conditional knockdown approaches beyond this study's scope. While the Keio collection provides unparalleled screening coverage, three inherent constraints merit acknowledgment: (1) Exclusion of essential gene targets; (2) Potential fitness costs and pleiotropic effects from gene deletions; (3) Possible undetected secondary mutations. Our multi-tiered validation strategy—prioritizing complemented mutants with pathway enrichment—mitigates these limitations while delivering mechanistic insights into EP’s vulnerabilities.

### Key Gene-Phenotype Relationships and Mechanistic Hypotheses

#### leuD: Bridging Leucine Biosynthesis and Antibiotic Stress

The *leuD* gene encodes a subunit of isopropylmalate dehydratase, a key enzyme in leucine biosynthesis. Leucine auxotrophy in *leuD* mutants is well-documented, but our data suggest a broader role in EP susceptibility. Chen et al. [26] demonstrated that *leuD* deletion globally perturbs metabolic networks, including fatty acid biosynthesis and stress-response pathways. Leucine scarcity may impair translation recovery under EP-induced stress, as amino acid deprivation exacerbates ribosomal stalling caused by EP’s primary target, leucyl-tRNA synthetase (LeuRS). Furthermore, Weber and Jung [27] observed repression of leucine biosynthesis genes under osmotic stress, suggesting cross-talk between metabolic homeostasis and antibiotic susceptibility. We propose that *leuD* deficiency creates a metabolic bottleneck, amplifying EP’s translational inhibition by limiting leucine availability and destabilizing proteostatis.

#### rnb: RNA Processing and Stress Adaptation

RNase II, encoded by *rnb*, is a 3’→5’ exoribonuclease critical for mRNA turnover and stress adaptation. Awano et al. [28] linked RNase II to cold shock responses, while Bárria et al. [29] implicated it in osmotic stress adaptation via degradation of outer membrane protein mRNAs. The *rnb* mutant’s EP hypersusceptibility may stem from dysregulated stress-response transcripts. For example, impaired degradation of mRNAs encoding efflux pumps or membrane remodeling proteins could disrupt EP detoxification or cell envelope integrity. Additionally, RNase II’s role in ribosomal RNA processing [30] suggests that *rnb* deletion may synergize with EP’s translational inhibition by stalling ribosome assembly, a hypothesis supported by the co-susceptibility of ribosome biogenesis factors like *yhbY*.

Moreover, Rnb is involved in processing monocistronic *leuX* precursor tRNA. Mutant analyses demonstrate that RNase II processes the 3′ downstream sequences of the *leuX* precursor tRNA after initial cleavage by PNPase or RNase P [31, 32]. The EP sensitivity of the Δrnb mutant suggests a functional link to this tRNA maturation pathway.

#### trmU (mnmA): tRNA Modification and Translation Fidelity

The *trmU/mnmA* gene encodes a tRNA-specific 2-thiouridylase critical for wobble uridine modification (s^2^U34) in tRNAs. This modification stabilizes codon-anticodon pairing and ensures translational fidelity. Alobaidallah et al. [33] reported that *trmU* inactivation potentiates β-lactam/aminoglycoside synergy, while Tamae et al. [34] observed ciprofloxacin resistance in *trmU* mutants. Furthermore, Çöl et al. [20] found *trmU* mutants hypersusceptible to boric acid, consistent with our observations regarding EP. We hypothesize that s^2^U34 deficiency alters tRNA structure, rendering LeuRS more susceptible to EP inhibition. Alternatively, defective tRNA modification may exacerbate mistranslation under EP stress, overwhelming quality-control systems such as the stringent response or proteasomal degradation.

#### ubiG: Ubiquinone Biosynthesis and Redox Imbalance

UbiG, an O-methyltransferase in ubiquinone (UQ) biosynthesis, catalyzes a penultimate step in the pathway. UQ is vital for electron transport and redox homeostasis. Søballe and Poole [35] linked *ubiG* mutants to oxidative stress susceptibility due to UQ intermediate accumulation, and Sevin and Sauer [36] reported increased osmotic stress sensitivity in these mutants.

The hypersusceptibility of *ΔubiG* mutants to EP aligns with established mechanisms linking UQ deficiency to redox imbalance. As UQ is essential for electron transport in aerobic respiration, its loss (e.g., in *ubiG* mutants) may lead to ROS accumulation due to impaired respiratory chain function [35]. EP may exploit this redox vulnerability by inducing reactive oxygen species (ROS) via LeuRS inhibition. Alternatively, UQ deficiency may impair ATP synthesis, hindering energy-dependent EP efflux or activation of stress responses. The pleiotropic metabolic effects of *ubiG* deletion underscore UQ’s centrality in bacterial resilience.

#### yhbY: Ribosome Biogenesis and Translational Collapse

YhbY, a ribosome assembly factor, facilitates the maturation of the 30S and 50S ribosomal subunits. Gagarinova et al. [37] reported genetic interactions between *yhbY* and RNases involved in rRNA processing. Since EP inhibits LeuRS and thus translation elongation, yhbY deficiency—by impairing ribosome assembly—can create a"dual hit"on protein synthesis. This combination may overwhelm compensatory mechanisms such as ribosome hibernation or stress granule formation, leading to translational collapse.

Furthermore, Lrp (Leucine-responsive regulatory protein) is a DNA-binding transcriptional regulator that represses *yhbY* transcription, and L-leucine binding to Lrp prevents this inhibition, enabling *yhbY* expression [38]. Because EP blocks leucine incorporation into tRNA^Leu^ by targeting LeuRS, this pathway links intracellular leucine levels to *yhbY* transcription. This explains the EP sensitivity of the *ΔyhbY* mutant.

### Converging Pathways Underpinning EP Sensitivity

**Fig. 7:**
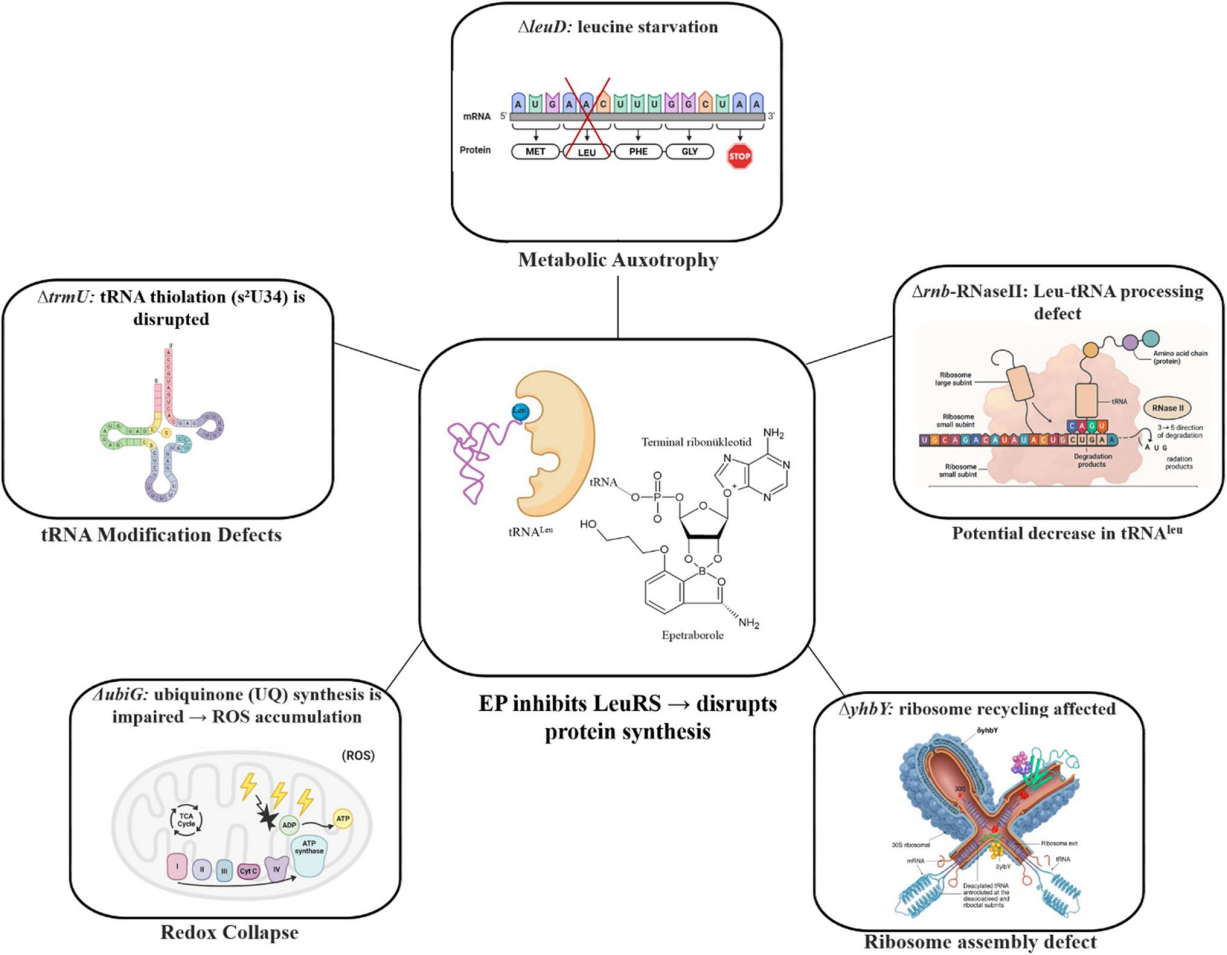
Mechanistic basis of EP sensitivity in *E. coli* mutants. This schematic illustrates the diverse cellular and molecular mechanisms contributing to hypersusceptibility to EP in specific *E. coli* mutants. At the center of the model is the inhibition of leucyl-tRNA synthetase (LeuRS) by EP, which blocks the aminoacylation of tRNA^Leu^ and disrupts protein synthesis. Surrounding this core are five functionally distinct mutant pathways: *ΔleuD* (top-center); loss of *leuD* disrupts leucine biosynthesis, resulting in leucine auxotrophy and impaired translation at leucine codons due to limited intracellular leucine availability. *Δrnb* – RNase II (top-right); deletion of *rnb* affects RNA turnover and tRNA maturation, potentially decreasing functional tRNA^Leu^ pools due to defective 3′ end processing. *ΔyhbY* (bottom-right); loss of the ribosome biogenesis factor YhbY impairs ribosomal subunit maturation, exacerbating translation defects under EP-induced stress. *ΔubiG* (bottom-left), deletion of *ubiG* disrupts ubiquinone biosynthesis, leading to redox imbalance and accumulation of reactive oxygen species (ROS), which compromise energy production and stress adaptation. *ΔtrmU* (top-left); loss of *trmU* impairs tRNA wobble uridine thiolation, reducing translation fidelity and potentially affecting the functionality of tRNA^Leu^. Together, these mutations converge on a central theme: disruption of tRNA^Leu^ function and translational capacity, which synergize with EP-mediated LeuRS inhibition to severely compromise bacterial viability

Based on the EP-hypersusceptible gene knockout mutants for which complementation was successful, our findings highlight multiple converging cellular pathways that sensitize *E. coli* to EP. By identifying gene deletions affecting amino acid biosynthesis, tRNA maturation/modification, redox balance, and ribosome function, we reveal a network of vulnerabilities that exacerbate the consequences of LeuRS inhibition (Fig. 7).

The *ΔleuD* mutant, lacking a key enzyme in leucine biosynthesis, emphasizes the importance of metabolic homeostasis in resisting EP-induced stress. Without endogenous leucine production, cells depend entirely on exogenous supply, heightening sensitivity to disruptions in leucyl-tRNA charging. Similarly, deletions in *rnb* (RNase II) and *trmU* impair tRNA stability and post-transcriptional modifications, both critical for maintaining a functional pool of tRNA^Leu^. Disruption of these processes likely compromises the availability and charging efficiency of tRNA^Leu^, intensifying the inhibitory effects of EP.

Strikingly, *ΔubiG* mutants—deficient in ubiquinone biosynthesis—accumulate ROS, showing that oxidative stress further sensitizes bacteria to translational inhibitors. Redox imbalance may disrupt energy production and macromolecular repair, reducing cellular tolerance to translation stress. Finally, deletion of *yhbY*, a ribosome assembly factor, suggests that impaired ribosome turnover magnifies susceptibility to LeuRS inhibition, particularly under conditions of aminoacylation stress (Fig. 7).

Collectively, these results demonstrate that EP sensitivity stems not only from direct inhibition of LeuRS but also from disturbances in pathways that preserve translational fidelity and stress resilience. These insights suggest that combination therapies targeting multiple nodes of the protein synthesis machinery and metabolic support systems could enhance the antibacterial efficacy of EP and related agents. Moreover, identifying such synthetic vulnerabilities offers a promising strategy for designing targeted antimicrobials that exploit specific metabolic and translational dependencies in bacteria.

### Systems-Level Analysis of EP Susceptibility

A comprehensive Omics Dashboard analysis was also performed to evaluate the genes associated with all the mutants susceptible to EP, and various biological systems and pathways were revealed. DAVID pathway analysis revealed that EP-susceptible mutants were involved in a number of cellular processes, including cellular response to UV, tRNA wobble position uridine thiolation, and sulfur transferase complex. In addition, STRING network analysis identified links between susceptible genes. In the following discussion, these findings are used to explore the known functions of specific genes in *E. coli* and their relationship to potential susceptibility mechanisms.

#### Omics Dashboard: Functional Clustering of Susceptibility Determinants

Categorization of EP-susceptible mutants revealed enrichment in: “Regulation” (37 genes), which includes sigma factor regulators (e.g., *rpoS*), transcription factors (e.g., *cusR*), and signaling kinases (e.g., *envZ*), “Central Dogma” (21 genes), spanning tRNA modification (*trmU*), RNA degradation (*rnb*), and ribosome biogenesis (*yhbY*), “Cell Exterior” (14 genes), encompassing transporters (*artJ*, *cmtA*) and membrane biosynthesis enzymes, and “Response to Stimulus” (14 genes), including stress sensors (*otsA*, *uspE*) and redox regulators (*ubiG*). (Fig. 4, Supplementary Table).

Among the EP susceptible gene hits in the categories of Regulation and Central Dogma, *artJ* gene, which is involved in arginine transport, was found to be up-regulated after carvacrol treatment [39]. Another study reported that cefiderocol resistance in an *E. coli* isolate was caused by mutations in the *cirA* gene, which is involved in iron transport [40]. Additionally, a study was conducted to investigate the mechanisms by which an NDM-producing *E. coli* isolate leads to resistance to cefiderocol and the combination of ceftazidime-avibactam and aztreonam. Whole genome sequencing was performed to identify resistance mechanisms, and frameshift mutations in the *cirA* iron transport gene were identified [41]. The study of the transcriptional response of *E. coli* to copper exposure employed a combination of DNA microarray analysis and *in vivo* and *in vitro* transcription assays. The findings of the study indicated that copper-responsive genes are subject to regulation by a hierarchy of regulatory networks, including CusR, which has been observed to form at least four regulators [42]. In a subsequent study, the investigation focused on the bacterial adaptation and defense mechanisms against silver. It was observed that a *cusR* mutant strain exhibited susceptibility to silver (0.4 +/- 0.1%) [43]. EnvZ has been identified as a regulator of biofilms and stress response expression [44]. A study demonstrated that OmpR-mediated expression of OmpC plays a significant role in ethanol tolerance through the EnvZ-dependent phosphotransfer signaling pathway [45]. Furthermore, it has been reported that combination mutations of *mrdA*, *envZ* and *ftsI* genes can increase resistance to imipenem (IMP) [46]. RT-PCR analysis revealed that exposure of *E. coli* cells to non-acidic stress conditions, encompassing alkalinity, oxidative stress (H₂O₂) and high salinity (NaCl), resulted in an augmentation of mRNA levels of *ydfD* when compared to standard growth conditions (pH 7, NaCl 1% and absence of oxidative stress) [47].

In “Response to Stimulus”, the analysis revealed that *envZ* [48], *loiP* [49], *otsA* [50] and *ubiG* [51] mutants were involved in the response to osmotic stress. The literature indicates that *uspE* and *hslO* are involved in the cellular response to oxidative stress [52, 53]. *ybcM* and *otsA* mutants were characterized in the literature as playing a role in the response to DNA damage [54].

In “Biosynthesis”, “Cell Exterior” and “Other Pathways”, bioinformatic analysis has revealed that some of the EP-susceptible gene hits are involved in different biosynthesis pathways in the cell. These include the biosynthesis of “cofactors, transporters and vitamins” (*pncA* and *ubiG*); the “biosynthesis of carbohydrates” (*fbaB* and *otsA*); and the “biosynthesis of metabolic regulators” (*otsA*) (Fig. 4, Supplementary Table). The analysis indicated that the susceptible gene hits, ArtJ and CmtA are transport proteins. ArtJ is a periplasmic binding protein that constitutes a component of an ABC transporter complex, which is involved in the transport of L-arginine into the bacterial cell [55]. The *cmtA* gene was demonstrated to play a role in the process of mannitol transport within the bacterial cell [56]. Bioinformatic analysis revealed that EP-susceptible mutants *trmU*, *mnmC*, *rnb* and *tusC* are involved in “macromolecular modification”, while *nuoL* is involved in “inorganic nutrient metabolism” (Fig. 4, Supplementary Table).

Furthermore, DAVID pathway enrichment analysis highlighted three key processes: (1) Cellular response to UV, which implicates DNA repair systems (e.g., *ybcM*), suggesting EP may indirectly induce DNA damage via ROS, (2) tRNA wobble uridine thiolation, which directly ties to *trmU/mnmA* and tRNA modification, aligning with EP’s LeuRS inhibition, and (3) Sulfur transferase complex, which suggests sulfur metabolism (e.g., Fe-S clusters) as a collateral target, potentially linking redox stress to EP susceptibility (Fig. 5).

In addition, STRING Network Analysis was conducted to evaluate functional modules and crosstalk (Fig. 6). Protein-protein interaction networks revealed two key clusters namely, Regulatory Hub (*rssB*, *envZ*, *cusR*, *creC*) and tRNA Modification Module (*mnmA*, *mnmC*, *tusC*). The genes *rssB*, *envZ*, *cusR*, and *creC* orchestrate stress adaptation (e.g., EnvZ-OmpR in osmotic responses [57], CusR in copper/silver detoxification [58]. EP may dysregulate these pathways, impairing cross-stress protection. The genes *mnmA*, *mnmC*, and *tusC* reinforces the centrality of tRNA thiolation [59, 60] and in terms of EP susceptibility, these genes may act possibly by modulating LeuRS activity or tRNA availability.

In summary, our findings position EP as a multi-faceted stressor targeting not only LeuRS but also interconnected processes. These include: Metabolic Vulnerability (*leuD* and *ubiG* mutants highlight the lethality of combining EP with metabolic disruption), Translational Inflexibility (*rnb*, *trmU*, and *yhbY* deficiencies exacerbate EP’s primary effect on translation, suggesting that inhibitors of RNA processing or ribosome assembly could synergize with EP) and finally Redox Imbalance (UQ biosynthesis defects (*ubiG*) and oxidative stress-susceptible mutants (*hslO*) imply EP induces secondary redox stress, a vulnerability exploitable by ROS-inducing adjuvants.

Furthermore, the therapeutic potential of EP against *Mycobacterium sp*., as demonstrated by Ganapathy et al. [13], prompted an investigation into conserved molecular targets through comparative sequence analysis. We performed BLASTP analyses to identify homologs of *E. coli* LeuRS and hypersusceptibility-associated genes. BLASTP (NCBI) revealed 48% identity (64% similarity) between *M. abscessus* LeuRS (GenBank: SHS77295) and its *E. coli* counterpart, indicating structural conservation. Homologs of LeuD, ArtJ, MnmA (TrmU), PncA, UbiG, and Rnb showed 49%, 31%, 34%, 39%, 41%, and 27% sequence similarity to Mycobacterium orthologs, respectively. This conservation suggests EP may exploit analogous vulnerabilities in Mycobacteria, particularly in tRNA modification and redox homeostasis pathways. Functional studies support this hypothesis. Notably, deletion of *mnmA* in *M. tuberculosis* abolished tRNA thiolation and attenuated intramacrophage growth, demonstrating its essential role in virulence [61]. Similarly, *leuD* deletion in *Mycobacterium avium subsp. paratuberculosis* (MAP) induces systemic metabolic dysfunction, including loss of nutrient utilization capacity and transcriptomic dysregulation affecting >100 genes under stress conditions [26]. These observations collectively suggest that EP's efficacy against Mycobacteria may stem from its ability to disrupt evolutionarily conserved nodes in central metabolic networks, particularly those governing translation fidelities through LeuRS inhibition, tRNA modification (MnmA/LeuD) and cellular redox balance (UbiG).

## Conclusion

This study identifies *E. coli* genetic determinants of epetraborole hypersusceptibility, implicating leucine biosynthesis, tRNA modification, RNA turnover, redox balance, and ribosome biogenesis as critical pathways. The convergence of these hits on translation and stress adaptation underscores EP’s capacity to amplify endogenous bacterial vulnerabilities. Future work should elucidate EP’s indirect effects on Fe-S clusters, ROS, and DNA damage, test EP combinations with inhibitors of tRNA modification or ribosome assembly and profile EP susceptibility in clinical isolates with mutations in orthologous genes. It should be emphasized that our study focused exclusively on the *E. coli* Keio collection, a defined model system that may not fully capture the genomic diversity and resistance mechanisms of clinical isolates. This limitation underscores the importance of future investigations profiling EP resistance across diverse multidrug-resistant pathogens, particularly Enterobacteriaceae and other priority Gram-negative species.

## Supplementary Information

ESM 1 (DOCX 2865 kb)

ESM 2 (DOCX 24 kb)

## Data Availability

No datasets were generated or analysed during the current study.
