# Supplementary material for: Genome-wide Screening of the Escherichia coli Keio Knockout Collection Identifies Genetic Determinants of Epetraborole Hypersusceptibility
Source: Eur J Clin Microbiol Infect Dis. 2025 Jun 13;44(9):2167–82. doi: 10.1007/s10096-025-05183-9 (PMC12457515; doi:10.1007/s10096-025-05183-9)
Supplement: Supplementary file 1 — (DOCX 2865 kb) [file 10096_2025_5183_MOESM1_ESM.docx]

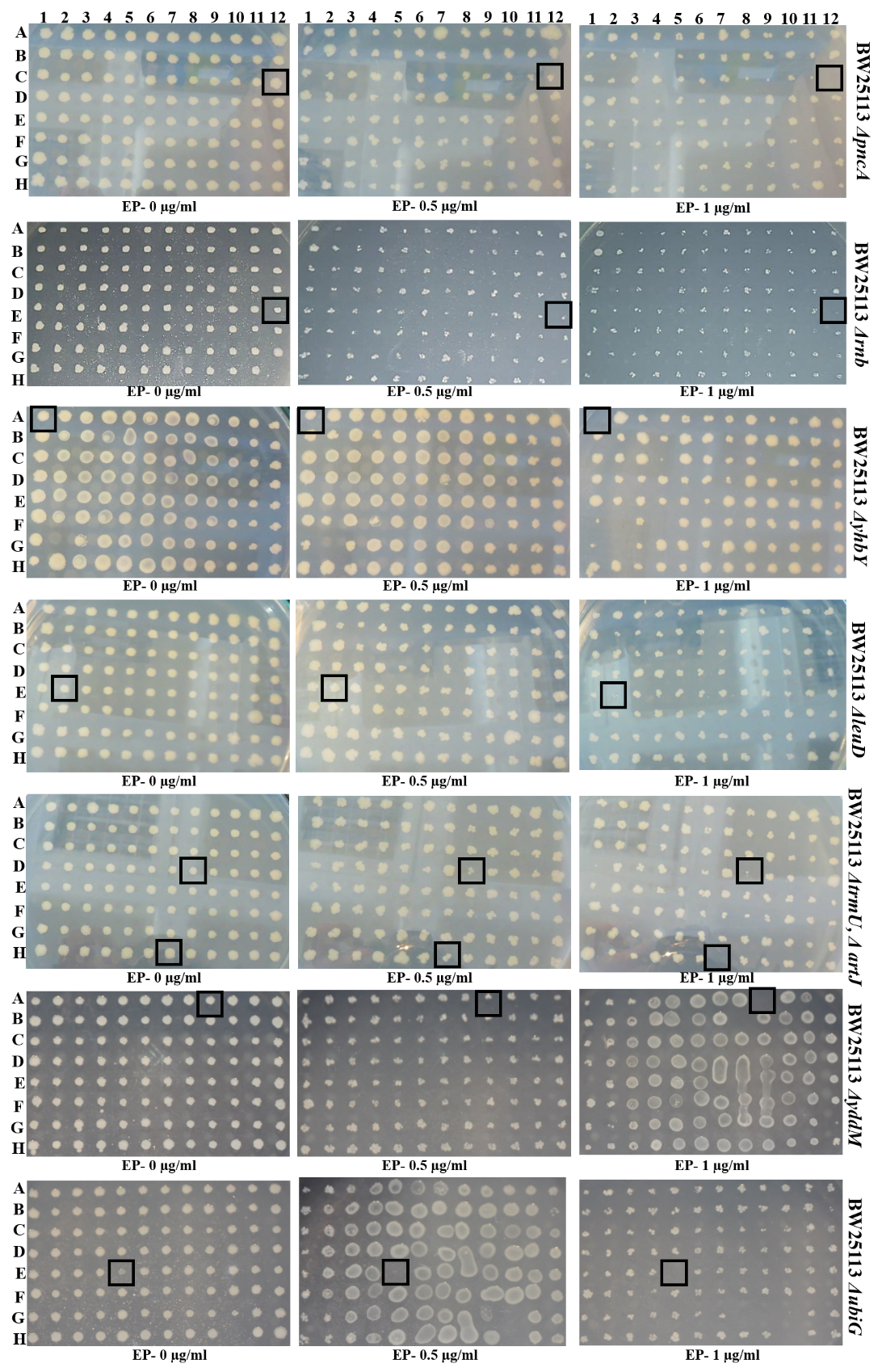


**Figure S1** Genome-wide screening identifies EP-hypersensitive E. coli mutants. Screening plates from the genome-wide EP susceptibility assay performed with E. coli K-12 BW25113 mutants from the Keio collection. Each row of images represents a mutant strain (ΔpncA, Δrnb, ΔyhbY, ΔleuD, ΔmntL, ΔartJ, ΔydhM, and ΔubiG), exposed to increasing concentrations of EP (0, 0.5, and 1 µg/ml). Hypersusceptible mutants exhibit reduced growth or complete growth inhibition at ≥0.5 or 1 µg/ml EP, as highlighted by black boxes. For instance, growth inhibition of the ΔpncA mutant is clearly observed at ≥1 µg/ml EP compared to surrounding colonies. These screening results were used to prioritize candidates for further validation and are representative of the full screening set (see Methods).


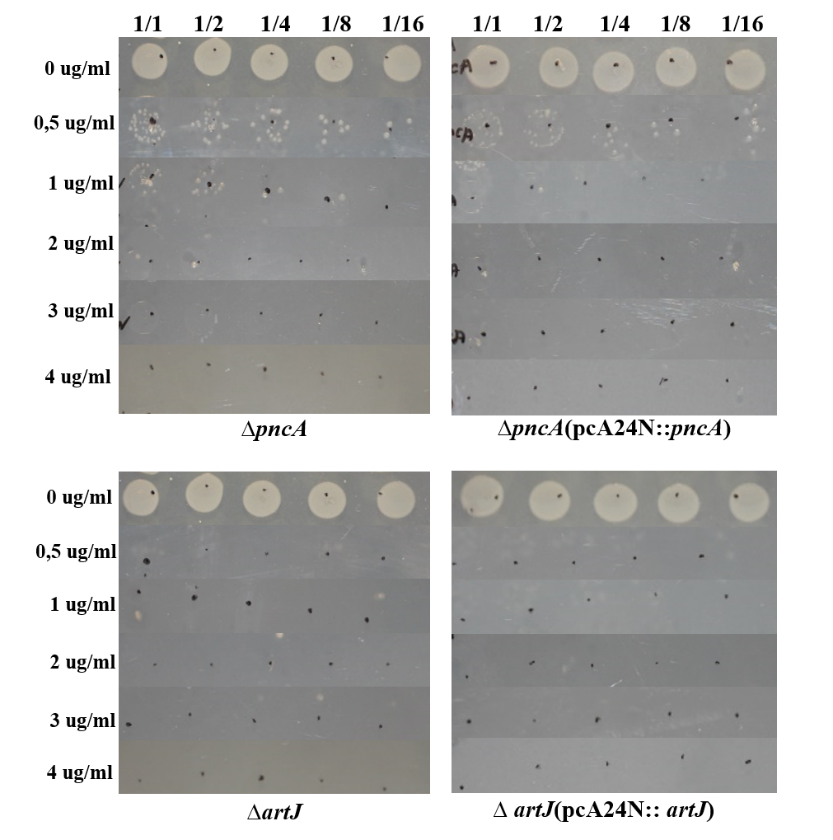


**Figure S2** Complementation analysis *ΔpncA* and *ΔartJ* mutants. Complementation restored EP resistance in *ΔubiG*, *ΔyhbY*, *Δrnb*, *ΔleuD*, and *ΔtrmU* mutants, confirming the involvement of these genes in resistance (see Figure 3). In contrast, complementation of *ΔpncA* and *ΔartJ* mutants using pCA24N::*pncA* and pCA24N::*artJ*, respectively, failed to restore resistance, suggesting that additional factors may be required or that expression from the plasmid was insufficient. Strains were serially diluted (1/1 to 1/16) and spotted onto agar plates as described in Materials and Methods. The strain names are shown on the figures. Spots seen indicate bacterial growth on LB agar plates containing EP concentrations (0–4 µg/ml).
