# Supplementary material for: Genome-wide Screening of the Escherichia coli Keio Knockout Collection Identifies Genetic Determinants of Epetraborole Hypersusceptibility
Source: Eur J Clin Microbiol Infect Dis. 2025 Jun 13;44(9):2167–82. doi: 10.1007/s10096-025-05183-9 (PMC12457515; doi:10.1007/s10096-025-05183-9)
Supplement: Supplementary file 2 — (DOCX 24 kb) [file 10096_2025_5183_MOESM2_ESM.docx]

**Supplementary Table.** Identification of EP susceptible gene mutants of *E. coli* through genome-wide screening of Keio mutant line and organization of the gene hits into system and subsystem using Omics Dashboard Analysis

| **System** | **Subsystem** | **Gene ID** | **Gene** | **Product** | |
| --- | --- | --- | --- | --- | --- |
| **Biosynthesis** | Cofactor, Carrier, and Vitamin Biosynthesis | EG11135 | *pncA* | nicotinamidase | |
|  | |  | EG11143 | *ubiG* | bifunctional 3-demethylubiquinone-8 3-O-methyltransferase and 2-octaprenyl-6-hydroxyphenol methylase |
|  |  | Carbohydrate Biosynthesis | G7129 | *fbaB* | fructose-bisphosphate aldolase class I |
|  |  |  | EG11751 | *otsA* | trehalose-6-phosphate synthase |
|  |  | Metabolic Regulator Biosynthesis | EG11751 | *otsA* | trehalose-6-phosphate synthase |
| **Degradation** |  | G7596 | *patA* | putrescine aminotransferase | |
|  | |  | G7129 | *fbaB* | fructose-bisphosphate aldolase class I |
| **Energy** |  | G7129 | *fbaB* | fructose-bisphosphate aldolase class I | |
|  | |  | EG12092 | *nuoL* | NADH:quinone oxidoreductase subunit L |
| **Other Pathways** | Enzymes not in Pathways | G7553 | *yghU* | disulfide reductase / organic hydroperoxide reductase | |
|  | |  | G7105 | *wzc* | protein-tyrosine kinase |
|  |  |  | G7237 | *frc* | formyl-CoA transferase |
|  |  |  | EG11291 | *loiP* | metalloprotease |
|  |  |  | G7201 | *epmC* | EF-P-Lys34 hydroxylase |
|  |  | Macromolecule Modification | EG11344 | *mnmA* | tRNA-specific 2-thiouridylase |
|  |  |  | EG11620 | *rnb* | RNase II |
|  |  |  | G7199 | *mnmC* | fused tRNA 5-aminomethyl-2-thiouridylate methyltransferase and tRNA 5-aminomethyl-2-thiouridylate synthase |
|  |  |  | G7713 | *tusC* | sulfurtransferase complex subunit |
|  |  | Inorganic Nutrient Metabolism | EG12092 | *nuoL* | NADH:quinone oxidoreductase subunit L |
| **Central Dogma** | Protein Metabolism | EG10269 | *envZ* | sensor histidine kinase | |
|  | |  | EG10730 | *creC* | sensory histidine kinase |
|  |  |  | G7105 | *wzc* | protein-tyrosine kinase |
|  |  |  | EG10889 | *rpmE* | 50S ribosomal subunit protein L31 |
|  |  |  | EG11291 | *loiP* | metalloprotease |
|  |  |  | EG12121 | *rssB* | RssB-P |
|  |  |  | G7201 | *epmC* | EF-P-Lys34 hydroxylase |
|  |  | DNA Metabolism | G6112 | *rayT* | REP-associated tyrosine transposase |
|  |  | RNA Metabolism | G6319 | *cusR* | DNA-binding transcriptional activator CusR-phosphorylated |
|  |  |  | G6195 | *prpR* | DNA-binding transcriptional dual regulator |
|  |  |  | EG12794 | *yhbY* | ribosome assembly factor |
|  |  |  | EG11344 | *mnmA* | tRNA-specific 2-thiouridylase |
|  |  |  | EG11620 | *rnb* | RNase II |
|  |  |  | EG10200 | *cytR* | DNA-binding transcriptional repressor |
|  |  |  | G7199 | *mnmC* | fused tRNA 5-aminomethyl-2-thiouridylate methyltransferase and tRNA 5-aminomethyl-2-thiouridylate synthase |
|  |  |  | EG11527 | *narP* | DNA-binding transcriptional dual regulator NarP-phosphorylated |
|  |  |  | G6302 | *ybcM* | DLP12 prophage; putative DNA-binding transcriptional regulator |
|  |  |  | EG10800 | *purR* | DNA-binding transcriptional repressor PurR |
|  |  |  | G7713 | *tusC* | sulfurtransferase complex subunit |
|  |  |  | EG12121 | *rssB* | RssB-P |
|  |  |  | G6774 | *yddM* | DNA-binding transcriptional regulator |
|  |  | Protein Folding | G7744 | *hslO* | molecular chaperone Hsp33 |
|  |  | Translation Proteins | EG10889 | *rpmE* | 50S ribosomal subunit protein L31 |
| **Regulation** | Sigma Factor Regulons | EG10269 | *envZ* | sensor histidine kinase | |
|  | |  | G7596 | *patA* | putrescine aminotransferase |
|  |  |  | EG11303 | *ydfD* | Qin prophage; lysis protein |
|  |  |  | EG10730 | *creC* | sensory histidine kinase |
|  |  |  | G6319 | *cusR* | DNA-binding transcriptional activator CusR-phosphorylated |
|  |  |  | G6195 | *prpR* | DNA-binding transcriptional dual regulator |
|  |  |  | EG11108 | *ybgA* | DUF1722 domain-containing protein |
|  |  |  | EG11628 | *artJ* | L-arginine ABC transporter periplasmic binding protein |
|  |  |  | G7105 | *wzc* | protein-tyrosine kinase |
|  |  |  | G6841 | *ynfB* | DUF1283 domain-containing protein |
|  |  |  | G7744 | *hslO* | molecular chaperone Hsp33 |
|  |  |  | G7129 | *fbaB* | fructose-bisphosphate aldolase class I |
|  |  |  | EG11620 | *rnb* | RNase II |
|  |  |  | EG10200 | *cytR* | DNA-binding transcriptional repressor |
|  |  |  | EG11143 | *ubiG* | bifunctional 3-demethylubiquinone-8 3-O-methyltransferase and 2-octaprenyl-6-hydroxyphenol methylase |
|  |  |  | EG10889 | *rpmE* | 50S ribosomal subunit protein L31 |
|  |  |  | EG11527 | *narP* | DNA-binding transcriptional dual regulator NarP-phosphorylated |
|  |  |  | EG10800 | *purR* | DNA-binding transcriptional repressor |
|  |  |  | EG12092 | *nuoL* | NADH:quinone oxidoreductase subunit L |
|  |  |  | G7237 | *frc* | formyl-CoA transferase |
|  |  |  | EG11291 | *loiP* | metalloprotease |
|  |  |  | EG11751 | *otsA* | trehalose-6-phosphate synthase |
|  |  |  | EG12121 | *rssB* | RssB-P |
|  |  |  | EG10155 | *cirA* | iron-catecholate outer membrane transporter |
|  |  | Signal transduction pathways | EG10269 | *envZ* | sensor histidine kinase |
|  |  |  | EG10730 | *creC* | sensory histidine kinase |
|  |  |  | G6319 | *cusR* | DNA-binding transcriptional activator CusR-phosphorylated |
|  |  |  | EG11527 | *narP* | DNA-binding transcriptional dual regulator NarP-phosphorylated |
|  |  | Transcription Factor Regulons | EG11792 | *cmtA* | mannitol-specific PTS enzyme IICB component |
|  |  |  | EG10269 | *envZ* | sensor histidine kinase |
|  |  |  | G7596 | *patA* | putrescine aminotransferase |
|  |  |  | EG11303 | *ydfD* | Qin prophage; lysis protein |
|  |  |  | G7026 | *yecT* | protein |
|  |  |  | EG10730 | *creC* | sensory histidine kinase |
|  |  |  | G6319 | *cusR* | DNA-binding transcriptional activator CusR-phosphorylated |
|  |  |  | G6195 | *prpR* | DNA-binding transcriptional dual regulator |
|  |  |  | EG12794 | *yhbY* | ribosome assembly factor |
|  |  |  | EG11108 | *ybgA* | DUF1722 domain-containing protein |
|  |  |  | EG11628 | *artJ* | L-arginine ABC transporter periplasmic binding protein |
|  |  |  | G7105 | *wzc* | protein-tyrosine kinase |
|  |  |  | G7189 | *yfbV* | PF04217 family membrane protein |
|  |  |  | G6841 | *ynfB* | DUF1283 domain-containing protein |
|  |  |  | G7129 | *fbaB* | fructose-bisphosphate aldolase class I |
|  |  |  | EG11344 | *mnmA* | tRNA-specific 2-thiouridylase |
|  |  |  | G7652 | *ubiU* | ubiquinone biosynthesis protein |
|  |  |  | EG10200 | *cytR* | DNA-binding transcriptional repressor |
|  |  |  | EG11143 | *ubiG* | bifunctional 3-demethylubiquinone-8 3-O-methyltransferase and 2-octaprenyl-6-hydroxyphenol methylase |
|  |  |  | EG11605 | *smg* | DUF494 domain-containing protein |
|  |  |  | G0-10445 | *ymjB* | ymjB |
|  |  |  | EG11527 | *narP* | DNA-binding transcriptional dual regulator NarP-phosphorylated |
|  |  |  | G8205 | *gapC* | gapC |
|  |  |  | EG10800 | *purR* | DNA-binding transcriptional repressor |
|  |  |  | G6355 | *ybeU* | DUF1266 domain-containing protein |
|  |  |  | EG12092 | *nuoL* | NADH:quinone oxidoreductase subunit L |
|  |  |  | G7237 | *frc* | formyl-CoA transferase |
|  |  |  | EG11246 | *uspE* | universal stress protein E |
|  |  |  | EG12121 | *rssB* | RssB-P |
|  |  |  | EG10155 | *cirA* | iron-catecholate outer membrane transporter CirA |
|  |  | Transcription Factors | G6319 | *cusR* | DNA-binding transcriptional activator CusR-phosphorylated |
|  |  |  | G6195 | *prpR* | DNA-binding transcriptional dual regulator |
|  |  |  | EG10200 | *cytR* | DNA-binding transcriptional repressor |
|  |  |  | EG11527 | *narP* | DNA-binding transcriptional dual regulator NarP-phosphorylated |
|  |  |  | EG10800 | *purR* | DNA-binding transcriptional repressor |
| **Response to Stimulus** | Proteins Involved in Response to Osmotic Stress | EG10269 | *envZ* | sensor histidine kinase | |
|  | |  | EG11143 | *ubiG* | bifunctional 3-demethylubiquinone-8 3-O-methyltransferase and 2-octaprenyl-6-hydroxyphenol methylase |
|  |  |  | EG11291 | *loiP* | metalloprotease |
|  |  |  | EG11751 | *otsA* | trehalose-6-phosphate synthase |
|  |  | Proteins Involved in Response to Heat | G7744 | *hslO* | molecular chaperone Hsp33 |
|  |  | Proteins Involved in Response to Oxidative Stress | G7744 | *hslO* | molecular chaperone Hsp33 |
|  |  |  | EG11246 | *uspE* | universal stress protein E |
|  |  | Proteins Involved in Response to DNA Damage | G6302 | *ybcM* | DLP12 prophage; putative DNA-binding transcriptional regulator |
|  |  |  | EG11751 | *otsA* | trehalose-6-phosphate synthase |
|  |  | Proteins Involved in Response to pH | G7237 | *frc* | formyl-CoA transferase |
|  |  | Proteins Involved in Response to Heat | EG11291 | *loiP* | metalloprotease |
|  |  | Proteins Involved in Response to Cold | EG11751 | *otsA* | trehalose-6-phosphate synthase |
|  |  | Other Proteins involved in Stimulus Response | EG10730 | *creC* | sensory histidine kinase |
|  |  |  | G6319 | *cusR* | DNA-binding transcriptional activator CusR-phosphorylated |
|  |  |  | G6195 | *prpR* | DNA-binding transcriptional dual regulator |
|  |  |  | EG12121 | *rssB* | RssB-P |
|  |  | Oxidant Detoxification Proteins | G7553 | *yghU* | disulfide reductase / organic hydroperoxide reductase |
|  |  | Other Proteins involved in Stimulus Response | EG11527 | *narP* | DNA-binding transcriptional dual regulator NarP-phosphorylated |
| **Cellular Processes** |  | EG11303 | *ydfD* | Qin prophage; lysis protein | |
| **Cell Exterior** | Transport Proteins | EG11792 | *cmtA* | mannitol-specific PTS enzyme IICB component | |
|  | |  | EG11628 | *artJ* | L-arginine ABC transporter periplasmic binding protein |
|  |  | Plasma Membrane Proteins | EG11792 | *cmtA* | mannitol-specific PTS enzyme IICB component |
|  |  |  | EG10269 | *envZ* | sensor histidine kinase |
|  |  |  | G7105 | *wzc* | protein-tyrosine kinase |
|  |  |  | G7189 | *yfbV* | PF04217 family membrane protein |
|  |  |  | EG11143 | *ubiG* | bifunctional 3-demethylubiquinone-8 3-O-methyltransferase and 2-octaprenyl-6-hydroxyphenol methylase |
|  |  |  | G6355 | *ybeU* | DUF1266 domain-containing protein |
|  |  |  | EG12092 | *nuoL* | NADH:quinone oxidoreductase subunit L |
|  |  |  | EG11940 | *yjcE* | putative transporter |
|  |  |  | G6496 | *yccF* | PF03733 family inner membrane protein |
|  |  |  | EG10730 | *creC* | sensory histidine kinase |
|  |  |  | EG11628 | *artJ* | L-arginine ABC transporter periplasmic binding protein |
|  |  | Periplasmic Proteins | EG10269 | *envZ* | sensor histidine kinase |
|  |  | Lipopolysaccharide Metabolism Proteins | G7105 | *wzc* | protein-tyrosine kinase |
|  |  | Outer Membrane Proteins | G6841 | *ynfB* | DUF1283 domain-containing protein |
|  |  |  | EG11291 | *loiP* | metalloprotease |
|  |  |  | EG10155 | *cirA* | iron-catecholate outer membrane transporter |

Article name: Genome-wide Screening of the *Escherichia coli* Keio Knockout Collection Identifies Genetic Determinants of Epetraborole Hypersusceptibility
